# Criminal Responsibility in the Insane

**Published:** 1894-10-13

**Authors:** 


### The Hospital

An Institutional Journal of

The Medical Sciences and Hospital Administration, with

AN EXTRA NURSING SUPPLEMENT.

Vol. XVII.—No. 420.] [Oct. 13, 1894.

### Criminal Responsibility in the Insane.

An interesting discussion and controversy has lately arisen on the criminal responsibility of persons affected in mind. It was begun at the recent meeting of the British Medical Association, and has been continued in the *British Medical Journal* and the *Times*. Law and medicine have differed in regard to this question during this century, and its expiration is evidently not to witness agreement. The questions at issue are these, A man commits a murder and is to be tried. Is the Court to take into account his state of mind before he is tried, or at all events before sentence is pronounced and executed, with the view of ascertaining in certain cases whether he was at the time the crime was committed in a state of mind to be held responsible for his actions? From a very ancient period, if the accused was manifestly insane or delirious, or completely imbecile, or was in the condition of a "wild beast," this state was held to bar his trial or to arrest the execution of the sentence. This common-sense procedure really yields the whole principle that science and medicine now contend for.

But what degree of mental disturbance or defect is necessary to exempt an accused person from criminal responsibility? Round this point has raged the chief controversy. The law has been, as was right, conservative of the essential principle of responsibility and amenability to punishment for crime. The safety and interests of society seemed to some of our greatest judges to be in peril if criminals were able successfully to plead irresponsibility for their actions on almost any grounds, and so to escape punishment. On the other hand, the study of disordered mental action in the brain, founded on an extended knowledge of its physiology, was continually advancing the line of irresponsibility for conduct and interference with self-control from brain disease into regions unsuspected before. The doctors were placed in the witness-box, and in regard to many criminals who were not manifestly delirous or imbecile they gave evidence that there was brain disease present which interfered with normal mental control. The legal and the popular instincts naturally resented this new departure. Again and again outcries from the most informed and influential journals were raised against the doctors, but especially against the "experts," and the "mad doctors." Perhaps individual doctors pushed their theories further than the facts warranted. But slowly and gradually the legal position swung towards the medical view.

Great legal luminaries, in the clearest language they could use, had laid down rules of law that were meant to be final, but these had to be given up. Lord Mansfield, in Bellingham's trial, admitted that the prisoner must know that his act was legally wrong to be held legally responsible. Then came the dictum of the whole of the judges in 1843, which has since that time been commonly regarded as the law of England. It was that an accused person must labour under such a mental defect as not to know the nature of the act he was doing, or that what he was doing was legally wrong, to be exempted from punishment for crime. Medicine never accepted this judicial hard and fast test of criminal responsibility. It held to this scientific position—Was the criminal act the result of disease or not? Had the accused normal power of action, or was his brain—the admitted vehicle of mind—in such a morbid state that his self-control was interfered with thereby?

It will be seen that the law set up a test of mere knowledge and of pure reason. Medicine laid down a principle of power over action, and of self-control. There has been much irrelevant assertion and counter-assertion on both sides, and a tendency to go to the logical limits of both positions, irrespective of the inherent difficulties of individual cases and the mystery of human motive, of individual mental idiosyncracy, and of "borderland" cases. A sharp controversy took place between the two keenest wits of both sides, Sir James Stephen and Dr. Maudsley, with no immediate result; but it helped to put the respective positions of the two professions in clear shape. Sir James Stephen, in his work on "Criminal Law," made a great surrender from the positions taken up by the judges of 1843. He practically yielded the scientific contention that if the criminal act of the accused was due to disease for which he was not directly responsible, then no punishable offence was committed. Several judges have applied Sir James Stephen's rule, and the legal test of knowledge alone may be said to be practically on its last legs. But the British Medical Association want the new position to be authoritatively defined by Parliament or by the Court of Appeal. No doubt in some way this end will be attained in no long time.

The next burning question is as to how the evidence as to the mental state of the accused is to be submitted to the Court. Our present British fashion is to allow the judge full power in this matter. The consequence is that one judge will not permit the expert to give any opinion or conclusion as to the mental condition of the accused, but only to state the facts of his interview, the conclusions from those facts being left to the jury; just as if the chemist in a poisoning case were to be asked about the arsenic found in the stomach of the victim, but must not be asked about the conclusion that this man was poisoned by it. An intense, almost childish, jealousy has been manifested against the purely fictitious idea of this danger of the accused being "tried by the experts" instead of by the judge and jury. Several distinguished judges have laid it down most forcibly, and their sentiments have been passionately re-echoed by some of the press, that the "plain man," by the aid of his common sense, is as good a judge of sanity or insanity as the most experienced psychiatrist. No doubt, in all questions of punishment and deprivation of civil status from insanity, the public is and must be supreme in its ultimate verdict. But on other questions medical knowledge must prevail.

Many of us would like to see such a change in the legal procedure as would provide for a more judicial and exhaustive expert opinion on the mental condition of an accused person by the appointment of skilled and experienced assessors to the Court instead of the present unsatisfactory method of experts being employed for each side, who have never consulted with each other previously and discussed the facts calmly and impartially as a scientific question. Such an improvement in the method of submitting evidence to the Court would at once put an end to the scandals of expert contradictions that sometimes occur. It would also put an end to the present almost ridiculous practice of two mental specialists being sent by the Home Secretary to examine the condemned man after he has been publicly tried and sentenced, and their unsworn "opinion" being then acted on in his future disposal irrespective of judge or jury! That may be "common sense," but it looks to plain men like a judicial farce.

Medicine in this, as in all else, appeals to the facts of nature. It meets with cases who know well that self-murder is wrong, morally and legally. Yet in obedience to an insane delusion, or to that "irresistible impulse" as to which the *Times* is so sceptical, commit suicide. Such cases are known to most men, and are never questioned. Murder is also committed under the same morbid influences. Why should this scientific fact be questioned? It is no more wonderful than suicide. Murder and suicide have been committed during somnambulism. Why should the study of such clinical facts and their explanation to a jury subject "experts" to scorn and sceptical derision? Is the brain and its mental functions in health and disease outside the pale of calm scientific treatment? The real fact is that the feeling against "experts" expressed by the *Times* is a remnant of the old prejudices in regard to insanity and everything connected with it. When will the reproach of madness cease?

